# TAM Plasticity under androgen deprivation therapy and PARP inhibition in prostate cancer: a multi-omics perspective

**DOI:** 10.3389/fimmu.2025.1745168

**Published:** 2025-12-18

**Authors:** Shuangming Chen, Weiwei Cai, Chunlin Liu

**Affiliations:** 1 Department of Urology, The Affiliated Taizhou Second People’s Hospital of Yangzhou University, Taizhou, Jiangsu, China; 2 Department of Interventional Radiology, The Affiliated Taizhou Second People’s Hospital of Yangzhou University, Taizhou, Jiangsu, China

**Keywords:** ADT/AR pathway inhibition, cGAS–STING signaling, multi-omics, PARP inhibitors, prostate cancer

## Abstract

Androgen deprivation therapy (ADT) and next-generation androgen receptor pathway inhibitors (ARPI) are increasingly combined with PARP inhibitors (PARPi) in metastatic prostate cancer (mPCa). These treatments have improved outcomes, yet responses remain variable and often lack durability. Single-cell and spatial multi-omics studies indicate that tumor-associated macrophages (TAMs) strongly influence therapeutic response and form a treatment-shaped continuum of states enriched for TREM2 and SPP1 programs, lipid metabolic activity, hypoxia adaptation, and phagocytic checkpoint signaling within the osteogenic cancer-associated fibroblast (CAF) niche. Macrophages also possess functional androgen receptor (AR) activity, which supports an AR-driven myeloid circuit that promotes immune exclusion during ADT or ARPI therapy. PARP inhibitors stimulate cGAS-STING and induce senescence-associated secretory phenotypes (SASP), leading to an initial type I interferon (IFN) response that ultimately transitions to an MDSC-like immunosuppressive phenotype. These processes converge on common mechanisms of phagocytic control through CD47-SIRPα, MerTK, Axl, CSF1R, and TREM2 and represent therapeutic targets for combination therapies. This review details the combined impact of ADT and PARPi and introduces a multi-omic framework that integrates TREM2 or SPP1 burden, STING activation status, phagocytic checkpoint expression, and HRR or SPOP genotype into a Myeloid Lymphatic Composite Score (MLCS). The MLCS is a scoring tool to assist in timing and selecting therapeutic combinations of ARPI with TREM2 or CSF1R blockade, PARPi with STING modulation, and ARPI with anti-CD47 therapy. Integrating mechanistic and translational data provides a foundation for biomarker-guided regimens capable of converting prostate cancer from an immune-cold disease to an immune-responsive state.

## 1 Introduction

In the past several years, androgen deprivation therapy (ADT) and next-generation androgen receptor pathway inhibitors (ARPI) when combined with poly (ADP-ribose) polymerase inhibitors (PARPi) have revolutionized the way we treat metastatic prostate cancer (mPCa) (1, 2). However, even with these recent developments in the field, the clinical response to treatment continues to exhibit significant variability across patients, as well as many patients only responding to therapy in the short term. One explanation for this phenomenon is that the tumor microenvironment (TME), particularly the presence of tumor-associated macrophages (TAM), is an important factor in determining a patient’s response to treatment and their subsequent recurrence of disease. Additionally, through recent advances in single-cell and spatial multi-omics profiling we have been able to redefine our understanding of TAM biology. TAMs no longer fit an outdated M1 or M2 binary framework but instead occupy a functional continuum defined by both lineage and microenvironmental context (3). This continuum is enriched for TREM2/SPP1 programs and immunosuppressive pathways (4, 5). These phenotypes are spatially co-localized with tumor cells, cancer-associated fibroblasts, and vascular niches, and are especially prominent within the osteogenic and immunologically excluded bone metastasis microenvironment in prostate cancer (6). In addition to tumor-inherent androgen signaling, myeloid cells themselves also express functional AR. Cioni et al. (7) demonstrated that AR activation in macrophages enhances TREM-1-mediated pro-tumor interactions and upregulates IL-10 and tissue residency markers.

Parallel to AR-directed therapy, PARP inhibition exerts significant immunomodulatory effects. PARPi activates cGAS-STING signaling and recruits immune cells within the prostate cancer bone metastatic niche. In a prostate cancer model, PARPi combined with PI3Ki can induce tumor regression by activating the cGAS/STING pathway within the TAM (8). However, treatment-induced cellular senescence and its associated secretory phenotype (SASP) can paradoxically remodel the tumor microenvironment in prostate cancer (9). And this “double-edged sword” effect may explain the heterogeneity of clinical efficacy of PARPi combined with immunotherapy (10).

These myeloid dynamics ultimately converge on phagocytic checkpoint pathways, including CD47-SIRPα and TREM2/CSF1R pathways (4, 11). Together, these axes determine the phagocytic recognition ‘starting line’ and the navigational route of antigens to T cells (12). Crossovers of these axes following ADT or PARPi provide opportunities for actionable combinatorial strategies, but they also will require biomarker-guided optimization of the timing and dosing of such therapies to prevent rebound myelopoiesis and excessive bone marrow suppression (13).

A multi-omics perspective that integrates scRNA and scATAC sequencing, spatial transcriptomics and proteomics, and liquid biopsy profiling can generate a mechanistic map linking treatment-induced signaling or secretory changes to TAM plasticity, T cell structure and function, and clinical outcomes. This enables composite biomarker development for patient stratification and adaptive trial design, such as TREM2 or SPP1 TAM ratios, STING activation indices, phagocytic regulatory balance metrics, and HRR or SPOP genotypes. This review synthesizes emerging mechanistic and translational evidence, elucidates how ADT and PARPi cooperatively reprogram TAM biology in prostate cancer, and proposes a biomarker-anchored myeloid-targeted combination therapy framework aimed at converting immunologically cold tumors into disease states with more durable therapeutic responses.

## 2 ADT driven myeloid reprogramming and the emerging AR myeloid axis

### 2.1 The functional AR myeloid axis and characterization of AR positive TAMs

Existing evidence suggests that androgen receptors (ARs) are functionally expressed in a subset of human macrophages. These receptors can reprogram inflammatory transcription, upregulate immune-regulatory mediators, and promote protumor cell–cell interactions beyond tumor epithelial cells, highlighting the existence of a genuine AR–myeloid axis to add to that of tumor epithelial cells (7). Drawing on this, single cell and mechanistic studies in prostate cancer highlight and AR^+^/TREM2^+^ inhibitory TAM state that becomes more prevalent in mPCa, and particularly in bone metastases. This state is characterized by gene modules such as IL10, TGFB1, and CCL2, which reinforce local immune rejection (4). Spatial profiling demonstrates that TREM2 or SPP1-labeled, AR-imprinted macrophages cluster near tumor nests, cancer-associated fibroblasts (CAFs), and vascular hubs. These microecological sites are linked to immune-excluded phenotypes and long-term treatment tolerance (4, 14). Conceptually, once epithelial AR signaling is disrupted by ADT or ARPI, tumors may increasingly rely on AR-modulated macrophages to sustain an inhibitory milieu, which may contribute to the limited efficacy and biochemical persistence of immune checkpoint inhibitors in unselected populations.

### 2.2 Cytokine and chemokine remodeling under ADT

ADT and next-generation AR pathway inhibitors remodel the cytokine and chemokine networks that govern monocyte recruitment and macrophage education. A central axis is CCL2–CCR2. Androgen blockade or AR loss increases CCL2 production from both tumor cells and stromal compartments, enhancing CCR2^+^ monocyte influx and downstream STAT signaling, which subsequently accelerates tumor progression and dissemination (15, 16). Concurrent alterations in the CXCL family (including CXCL2 and CXCL8 within inflammatory circuits, and CXCL14 within matrix niches), together with regulatory cytokines such as IL10 and TGFB, further drive macrophages toward an inhibitory phenotype (17, 18). The ADT-induced secretory program, which resembles a treatment-associated senescence-associated secretory phenotype (SASP), amplifies these myeloid-directed signals and creates a time-evolving window in which monocyte recruitment peaks before adaptive antitumor immunity can be established (19). The bone metastatic niche intensifies this effect. Osteoblasts, osteoclasts, CAF subsets, and endothelial cells collectively generate CCL2, diverse CXCL ligands, and growth factors. At the same time, hypoxia and increased access to lipids induce local TREM2 or SPP1 programs and reinforce dominance of phagocytic checkpoints (4, 14, 20). All in all, ADT is not immunologically inert. Rather, in the absence of appropriate combination strategies, it tips the chemokine network to myeloid accumulation and conditioning.

### 2.3 Spatial and single cell insights into metabolic adaptation and niche localization

TAM trajectories shaped across primaries and mPCa lesions revealed from scRNA and ATAC-sequencing and spatial transcriptomic/proteomic mapping. Pseudotime analyses show that AR inhibition pushes monocyte-derived macrophages toward lipid metabolic activity, hypoxia adaptation, and TREM2 or SPP1 dominance, with tafalizumab (anti-TREM2) and GSK3147 (blocking SPP1) suggesting impaired antigen presentation and facilitating T cell rejection (21, 22). Neighborhood analyses show TAM-tumor-CAF triads forming immunosuppressive hotspots whose higher density is associated with progression to metastasis and poor response to systemic therapy (22). These datasets represent measurable readouts of pharmacodynamics (TREM2 or SPP1 TAM ratio; spatial neighbor scores; ligand–receptor pairs such as APOE-TREM2 and CSF1-CSF1R) and can inform ADT-based combination therapy trials in defining pharmacologic response (22, 23).

### 2.4 Therapeutic implications for targeting the AR TAM circuit

Therapeutic implications follow directly from these circuits. First, combining ARPI with TREM2 blockade aims to disrupt the APOE–TREM2–AR positive feedback loop in macrophages, weaken inhibitory niche maintenance, and restore antigen flow toward T cells. Preclinical evidence indicates that this combination can reeducate macrophages and inhibit tumor progression (4). Second, ARPI combined with CSF1R inhibition reduces the survival and maintenance of target TAM subsets. Model studies suggest that such combinations can reverse resistance to androgen blockade, although dosing schedules must avoid prolonged bone marrow suppression and rebound myeloid hyperplasia upon discontinuation (24). Given that ADT transiently enhances chemokine-driven myeloid recruitment, treatment sequencing becomes critical. In the early phase following AR inhibition, when monocyte recruitment and macrophage education peak, myeloid-targeted therapies may achieve maximal benefit. In later phases, therapeutic emphasis may need to shift toward additional mechanisms, including phagocytic checkpoint regulation (25). Therefore, experimental designs should integrate spatial and multi-omics pharmacodynamic indicators, such as the reduction of TAM-tumor-CAF neighbor clusters, decreased TREM2 or SPP1 TAM load, and normalization of CCL2, IL10, and TGFB. These parameters should be considered in combination with rPFS and OS as a coherent primary endpoint set to account for both mechanistic effect and durability (5, 22). In conclusion, ADT is not a straightforward subtraction of epithelial AR signaling. It rewrites the cytokine and chemokine landscape, revealing a druggable AR-TAM axis that must be co-managed in order to change the disease from an immunologically cold to a conditionally hot disease state (**Table 1**).

**Table 1:** ADT reshapes the myeloid axis: AR-TAM crosstalk and plasticity (Chapter 2).

Key mechanism/findings	Biomarkers/signatures	Spatial/context	Clinical implications	PD readouts to track	Refs
Functional AR in Myeloid Cells: AR signaling in macrophages rewires inflammatory transcription and upregulates IL-10/TGF-β; promotes an AR^+^/TREM2^+^ inhibitory state that sustains immune exclusion even after epithelial AR suppression.	AR-myeloid targets; TREM2, SPP1, IL10, TGFB1, CCL2 signatures	AR-imprinted TREM2/SPP1 TAMs cluster near tumor nests, CAFs, and vasculature; specifically enriched in the bone metastatic niche.	Explains “immune-cold” phenotypes and limited efficacy of immune checkpoint inhibitors (CPI) in mPCa.	AR^+^/TREM2^+^/SPP1^+^ TAM fraction; TAM–tumor proximity scores; APOE–TREM2 pathway activity.	(4, 7, 14)
Chemokine Remodeling: ADT/ARPI triggers a surge in monocyte recruitment via CCL2–CCR2; alters CXCL family (CXCL2/8/14) and induces a SASP-like secretome that conditions macrophages toward inhibition.	CCL2, CCR2, CXCL2/8/14, IL-10, TGF-β; STAT3 activation	Bone Niche Context: Osteoblasts, osteoclasts, and endothelial cells amplify chemokine output; hypoxia and lipids reinforce TREM2/SPP1 programs.	ADT is not immunologically neutral; it creates a transient recruitment window that tips toward immunosuppression if uncontrolled.	Plasma CCL2, IL-10, TGF-β levels; CCR2^+^ monocyte count; tissue CXCL panel expression.	(4, 14–20)
Single-Cell & Spatial Trajectories: AR inhibition drives monocyte-to-macrophage differentiation toward lipid-metabolic, hypoxia-adapted, and TREM2/SPP1-dominant states; formation of “Spatial Triads”.	Trajectory-to-TREM2/SPP1 pseudotime scores; Lipid metabolism signatures; Hypoxia adaptation	TAM–Tumor–CAF Triads: High-density immunosuppressive hotspots (“neighbor clusters”) physically excluding T cells.	Identifies specific niches of resistance; suggests need for spatial pharmacodynamics in ADT combination trials.	TREM2/SPP1 TAM ratio; Spatial Neighbor Index (Triad density); Ligand-receptor pairs (APOE–TREM2, CSF1–CSF1R).	(14, 21–23)
Therapeutic Targeting: Combination strategies to break the loop. ARPI + TREM2 inhibition disrupts the APOE–TREM2–AR loop; ARPI + CSF1R reduces TAM survival. Sequence is critical (early vs. late).	High TREM2/SPP1 burden; Elevated CCL2/IL-10/TGF-β; Active APOE–TREM2 or CSF1–CSF1R signaling	Aim to collapse TAM–tumor–CAF spatial neighborhoods and reduce suppressive hotspots in bone lesions.	Target early post-ADT (peak recruitment) with myeloid agents; late phase requires phagocytic checkpoint control. Monitor marrow safety.	Reduction in TREM2/SPP1 TAM load; Collapse of spatial triads; Normalization of cytokines; Correlate with rPFS/OS.	(4, 5, 22, 24, 25)

## 3 PARP inhibition and innate immunity with the double edged effect of STING activation and SASP

### 3.1 Beyond synthetic lethality involving cGAS-STING activation and genotypic determinants

**Figure 1:**
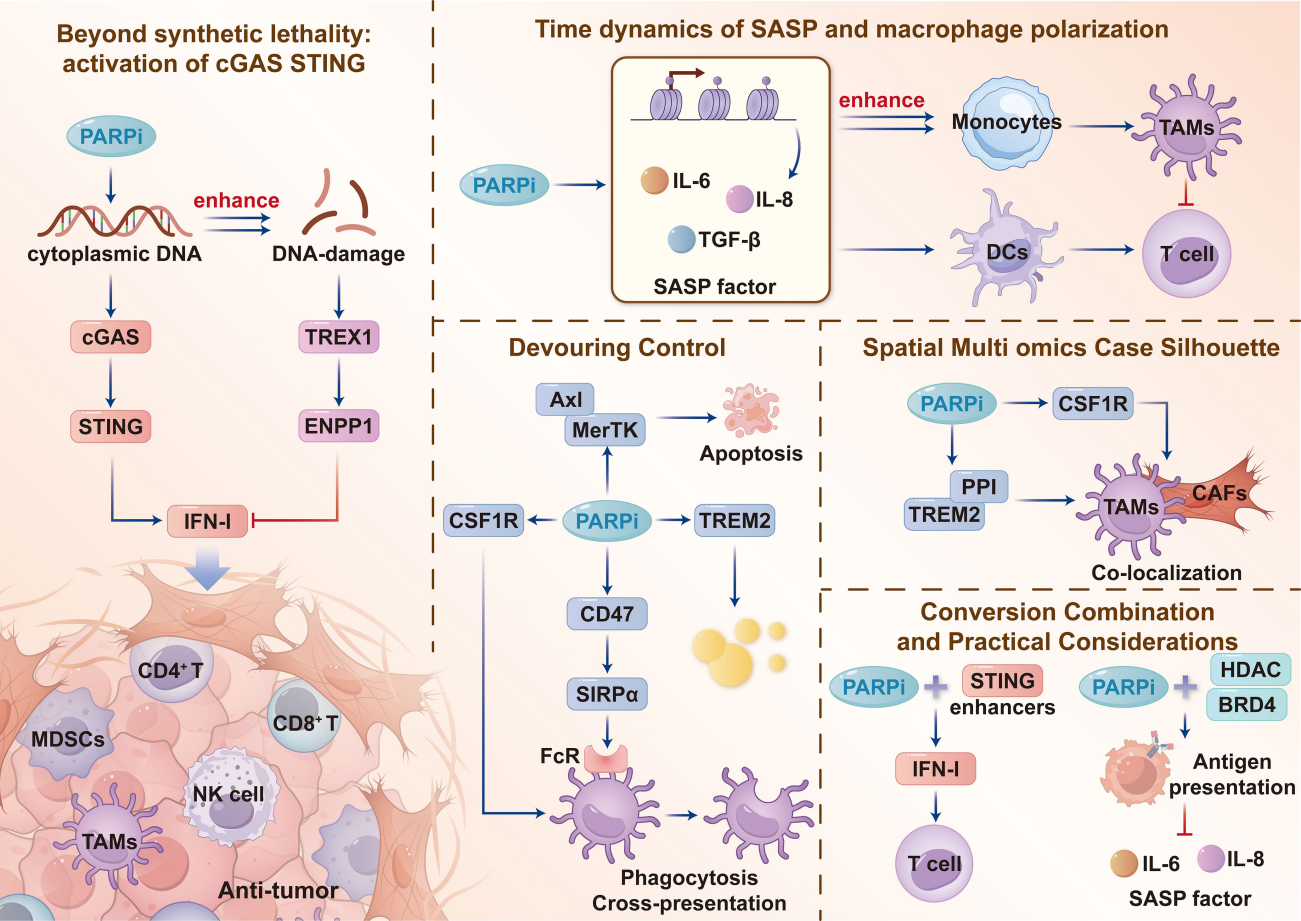
PARP inhibition–mediated immunomodulation and TAM reprogramming in prostate cancer. PARP inhibition (PARPi) induces cytoplasmic DNA accumulation and micronuclei, activating cGAS–STING to release Type I interferons (IFN-I) and recruit CD4^+^/CD8^+^ T cells. Negative regulators TREX1 and ENPP1 dampen this response. Concurrently, PARPi triggers a Senescence-Associated Secretory Phenotype (SASP) via IL-6, IL-8, and TGF-β, driving suppressive TAM polarization. Phagocytosis is restricted by the upregulation of CD47–SIRPα, MerTK, and TREM2 checkpoints (“Devouring Control”). Spatially, these shifts foster immunosuppressive TAM–CAF–tumor triads. Combinations with STING enhancers or epigenetic modulators (HDAC/BRD4 inhibitors) aim to extend the pro-inflammatory window and restore antigen presentation. PARPi, poly (ADP-ribose) polymerase inhibitor; cGAS, cyclic GMP-AMP synthase; STING, stimulator of interferon genes; IFN-I, type I interferon; TAM, tumor-associated macrophage; SASP, senescence-associated secretory phenotype; TREX1, three prime repair exonuclease 1; ENPP1, ectonucleotide pyrophosphatase/phosphodiesterase 1; CD47, cluster of differentiation 47; SIRPα, signal regulatory protein alpha; TREM2, triggering receptor expressed on myeloid cells 2; CSF1R, colony stimulating factor 1 receptor; CAF, cancer-associated fibroblast; HDAC, histone deacetylase; BRD4, bromodomain containing 4.

PARP inhibitors (PARPi) were initially developed to take advantage of HRR deficiencies and induce synthetic lethality, however, their immunological effects extend well beyond DNA repair. By trapping PARP on damaged chromatin and augmenting unresolved DNA lesions, PARP inhibitors promote the generation of micronuclei and cytoplasmic DNA in prostate cancer and instigate activation of the cGAS–STING pathway. This activation induces interferon-mediated responses and subsequently enhances natural killer cell and macrophage activity, as well as the infiltration, proliferation, and antitumor response of CD4 and CD8 T cells within tumors (**Figure 1**) (8, 26). The magnitude and quality of this response are context dependent. Prostate cancers with HRR deficiency, particularly those with germline mutations, display a more T cell–inflamed microenvironment than sporadic tumors; however, HRR-proficient prostate cancers are typically immunologically cold with substantial infiltration of immunosuppressive myeloid-derived suppressor cells (MDSCs) and tumor-associated macrophages (TAMs) (27, 28). Notably, cGAS–STING activation is time restricted and potentially self-limiting in prostate cancer. With sustained DNA-damage pressure, negative regulatory mechanisms such as TREX1 upregulation, which degrades cytosolic DNA, and ENPP1 activity, which hydrolyzes extracellular cGAMP, gradually emerge to suppress type I interferon production and promote immune escape (29, 30).

### 3.2 Temporal dynamics from STING driven inflammation to SASP mediated suppression

Beyond acute nucleic acid sensing, PARPi can induce treatment-related cellular senescence in tumor and stromal compartments, generating a senescence-associated secretory phenotype (SASP) enriched in IL6, IL8 or CXCL8, CCL2, GM-CSF, and members of the TGF beta family (31–33). The composition and timing of SASP are critical. Early SASP programs can cooperate with STING activation to enhance antigen acquisition by dendritic cells and promote T cell priming. In contrast, protracted SASP enhances monocyte attraction and macrophage polarization toward immune suppression and tissue repair, leading to exclusion of T cells and lack of treatment efficacy (34). In prostate cancer models, PARPi-conditioned media drive chemotaxis of CCR2 positive monocytes and SPP1- or TREM2-biased TAM polarization during exposure to hypoxic and lipid-rich microenvironments, characteristics of the bone metastatic microenvironment (14, 20, 35). Together these observations highlight a two-stage paradigm where an initial wave of STING-mediated inflammation is followed by a SASP-induced inhibiting drift unless ameliorative processes intervene.

### 3.3 Regulating phagocytosis and balancing CD47 with pro phagocytic signals in the bone niche

As macrophages infiltrate PARPi-treated tumors, phagocytic regulation becomes a critical determinant of the treatment effect in prostate cancer. The CD47-SIRPα axis delivers the canonical “don’t eat me” signal, and PARPi or other DNA-damage stressors can upregulate CD47 and related checkpoints, elevating the phagocytic threshold (36, 37). Effective reversal typically requires antibody-mediated opsonization with intact Fc effector function, or exposure to “eat me” cues such as calcium flux or phosphatidylserine externalization, to redirect macrophages from trogocytosis toward productive phagocytosis and cross-presentation (38). Parallel receptor systems further modulate this threshold. MerTK and Axl integrate apoptotic cell clearance with immunoregulatory outputs that often suppress antigen presentation; TREM2-positive macrophages accumulate under metabolic and hypoxic stress and favor lipid processing and tissue repair programs; CSF1R sustains TAM survival and repair-oriented states; and CXCR2 expressed on myeloid cells and neutrophils coordinates their chemotaxis toward DNA damage–associated inflammatory niches (39–41). PARPi-induced remodeling of the tumor microenvironment can upregulate one or more of these pathways, providing a mechanistic rationale for combining phagocytic checkpoint blockade (for example, anti-CD47 or anti-SIRPα) or myeloid-reprogramming agents (including CSF1R, TREM2, or MerTK inhibitors), and potentially modulators of antigen-processing enzymes like Cathepsin S, to convert macrophage accumulation into effective antitumor immunity in prostate cancer (42).

### 3.4 Spatial architecture of TAM tumor CAF triads and metabolic niches

In advanced and castration resistant prostate cancer, spatial transcriptomics and imaging mass spectrometry flow cytometry showed co localization of TREM2 ^+^/SPP1 ^+^ TAM with hypoxic and lipid rich tumor regions, adjacent blood vessels, and specific CAF subgroups (14). Proteomic and phospho-signaling analyses reveal activation of APOE–TREM2 and CSF1R pathways within these ecological niches, consistent with tissue repair–oriented and immunosuppressive TAM phenotypes (4). Metabolomic and lipidomic profiling further identifies signatures of cholesterol esterification and oxidative phosphorylation, and emerging metabolic vulnerabilities such as disulfidptosis, which align with the dominance of phagocytic checkpoint programs in these macrophage clusters (43, 44). Notably, following PARPi exposure, the spatial neighbor index representing TAM–tumor–CAF triads transiently expands and then contracts when effective myeloid-targeting combinations are applied. These dynamics offer measurable pharmacodynamic readouts for the particular case (TREM2 or SPP1 TAM ratio, APOE-TREM2 ligand-receptor activity, CD47 density maps) (4, 11, 14). For these reasons, it is often the case that the “where” and “who” macrophages interact with will predict the treatment fate as much as “how many” are there.

### 3.5 Combinatorial strategies for converting cold tumors via myeloid reprogramming

Mechanistic insights lead to other testable hypotheses for paradigms exploiting PARPi combination strategies that utilize such early innate immune activation at the expense of later more suppressive myeloid programs. One such strategy combines PARPi with STING enhancers-STING agonists or cGAS stabilizers that promote more vigorous early interferon signaling and T cell chemotaxis before the SASP-driven inhibition takes over. Preclinical data suggest that these combinations are compatible with stamping the antigen presenting cell licensing harder, but one cannot discount the cytotoxicity seen with systemic cytokines from such a combination (8, 45). Another such strategy is to combine PARPi with the epigenetic regulators of choice HDAC or BRD4 inhibitors, targeting axis such as SENP1-HDAC2 interactions, that may further support the viral mimicry programs or the antigen presentation at the same time as playing interference with the more protumorigenic SASP components to lengthen the pro-inflammatory window (46–48).

Another justification stems from pairing PARPi with anti-CD47 or anti-SIRPα therapies to reduce phagocytic thresholds and drive the additional myeloid presence towards effective tumor elimination and cross-priming of T cells. Efficacy will depend on development of Fc-engineered antibodies and precise timing, ideally post-peak recruitment of macrophages (49). There are also explorations pairing PARPi with inhibitors of CSF1R, TREM2, or MerTK, to disrupt TAM survival or lipid-repair programs, though these must be carefully titrated to avoid excessive marrow suppression and rebound myelopoiesis (4, 41). Upstream therapy such as VEGF blockade or radiotherapy pretreatment can also be matched to the PARPi activities to help with vasculature normalization or immunogenic cell death leading to better antigen access and improved innate-adaptive communication (50, 51). There are two design principles that emerge consistently. First, make sure that the sequences and timing are correct - the primary waves of intervention should be at its site of perception in the innate immune space, followed by recalibrating thresholds of ‘eating’. Second, inclusion of biomarker data is paramount; HRR or SPOP genotype, STING activation index, TREM2/SPP1 TAM ratio, CD47 burden, and distance to counting score could all be combined to largely form a selection criterion for ideal candidates and determine treatment windows (4, 14, 45, 49). Overlapping toxicities are inevitable, especially with myeloid-target agents, and being mindful of overlapping toxicities will be paramount in embedding space and multi-omics pharmacodynamic assessments into that trial design and implementing adaptive dosing strategies (13).

## 4 Integration of multiple omics approaches for patient stratification and combination design

### 4.1 Data layer and analysis process

The approach operationally stratifies prostate cancer in the context of ADT and PARPi which requires integration across multiple complementary omics layers: single-cell RNA-seq and ATAC-seq to resolve cell states and regulatory potential; spatial transcriptomics, proteomics, and imaging mass cytometry to preserve neighborhood context; proteomics and phosphoproteomics to capture pathway activity; metabolomics and lipidomics to identify hypoxia-driven lipid remodeling in TAM; and with liquid biopsy analytes, including cfDNA and exosomal profiles for longitudinal monitoring of tumors (52, 53). Recent studies have further demonstrated the power of integrating pan-cancer multi-omics and Mendelian randomization to identify robust causal biomarkers (54, 55). These modalities should be grounded to the specific pathology of mCRPC bone metastases. Single-cell analysis is then used to explicitly resolve the conversion of recruited monocytes into AR-driven or STING-activated myeloid states, while spatial transcriptomics and proteomics map these phenotypes onto the osteogenic and vascular “triads”. Metabolomic integration correlates hypoxia driven lipid accumulation with phagocytic checkpoint expression, all within the therapeutic biology of the bone niche.

One robust starting point for an analysis pipeline is a workflow that begins with correction of the data for batch-effect and alignment, followed by cell-state annotation using a macrophage and T-cell atlas that was previously developed to study prostate cancer bone metastasis (56, 57). Ligand-receptor inference tools that quantify interactions of tumor cells with CAFs and TAMs will be leveraged. Further, trajectory analysis might be utilized to model transitions of monocyte-derived macrophage to either TREM2 or SPP1 programs, as these might be elicited under different therapeutic pressures (14, 58). For multimodal integration, methods like MOFA+, totalVI, and multi-omics graph neural networks combine RNA, protein, and phospho-signatures with spatial features to generate patient-level descriptors for clinical modelling (52, 59). Quality control should be rigorous to capture the most important biological and technical factors in disease biology, including continuity along the myeloid lineage trajectory, T-cell depletion gradients, and covariates such as cell composition and tumor content in bone biopsies. Given the tendency towards overfitting in studies with small sample sizes, analyses should follow preregistered protocols and transparent model governance.

### 4.2 Defining the myeloid lymphatic composite score MLCS

We propose a practical biomarker framework for ADT and PARPi decision-making, termed the Myeloid Lymphatic Composite Score (MLCS). MLCS integrates four major components. The first is the TREM2 or SPP1 TAM ratio reflecting lipid-conditioned and repair-oriented macrophage states. The second is a STING activation signature represented by interferon-stimulated gene modules and TBK1 or IRF3 phosphorylation. The third is a phagocytosis index balancing CD47 burden with “eat me” signals and MerTK or Axl pathway activity. The fourth is tumor genotype, specifically HRR deficiency and SPOP mutation, which determines PARPi sensitivity and influences basal innate immune signaling (1, 4, 12, 60–63). MLCS thresholds can be optimized through nested cross-validation and calibrated to clinical endpoints such as rPFS and OS. Decision-curve analysis can then quantify net clinical benefit relative to existing stratification systems (64, 65). External validation should include multicenter retrospective spatial-omics datasets and prospective treatment-biopsy cohorts designed to test directional effects, for example whether ARPI combined with TREM2 blockade reduces TREM2 or SPP1 TAM programs and decreases spatial distance between TAM and effector T-cell neighborhoods (66, 67). To minimize platform drift, the MLCS system should be implemented using a locked feature dictionary with fixed gene and protein panels and image-derived metrics. Normalization must be performed relative to predefined reference controls, and unit-testing with standardized samples should confirm assay performance prior to clinical deployment (68, 69).

### 4.3 Clinical implementation using the three layer readiness panel

Given the technical challenges and low cellularity of bone metastasis biopsies in prostate cancer, the clinical version of MLCS should prioritize the smallest yet most robust readout set. We recommend a three-layer clinical readiness panel composed of the following elements. The first component, designated as the Tissue Layer, assesses myeloid burden by utilizing IHC or IF to quantify the density of TREM2^+^/SPP1^+^ macrophages and their spatial proximity to tumor nests, where a high score signifies a repair-oriented and immunosuppressive niche. This is enhanced by the Molecular Layer that assesses inflammation status by RNA panel of about 50 to 100 genes for measuring markers of STING activation (IFN-I signatures) and chemokine output (CXCL9, CXCL10). This molecular ‘classifies’ immunologically ‘cold’ tumors from ‘conditionally hot’ tumors. Finally, the Liquid Layer establishes systemic and genomic data through plasma proteomics for soluble CD47 + CSF1 detection and cfDNA genotyping for HRR deficiency or SPOP mutations to establishes baseline sensitivity to PARPi (4, 63, 70–72). For pelvic tumors, urinary exosomes may provide additional data, although metastatic castration-resistant prostate cancer (mCRPC) shows wide variability in exosome yield. In all sample types, strict adherence to pre-analytical SOPs is necessary since the type of biopsy needle, decalcification method and time to process plasma, for example, can all heavily affect signal stability and data quality (73). For broad clinical adoption, every assay should return an easy red-yellow-green readout and map it to combination therapy options: ARPI combined with anti-CD47 therapy in the case of an MLCS myeloid-high profile containing high CD47 burden, for example. Labs performing this work could function as LDTs and participate in proficiency testing (74, 75).

### 4.4 Biomarker guided trial design and adaptive strategies

Embedding MLCS into experimental approaches allows for rapid iterative learning–confirming cycles. Patient enrichment should include separate HRR-deficient and SPOP-mutant subgroups. In terms of mapping scores to specific treatments, the MLCS profile dictates the therapeutic strategy. Tumors exhibiting a high TREM2 or SPP1 burden alongside low STING activity are prioritized as candidates for ARPI combined with macrophage-targeted therapies, including anti-TREM2 or CSF1R inhibitors. Conversely, tumors characterized by a high CD47 burden and intermediate inflammation levels are selected for combinations involving phagocytic checkpoint inhibitors, such as anti-CD47 therapy, to lower the threshold for tumor clearance (2, 5, 76–78). Adaptive randomization can allocate patients across multiple treatment groups such as ARPI with TREM2 inhibition, PARPi with STING enhancement, or ARPI with anti-CD47 therapy. Early pharmacodynamic thresholds should be predefined, for example a reduction of thirty percent or more in the TREM2 or SPP1 TAM ratio at week six, accompanied by the collapse of TAM–tumor–CAF neighbor clusters, as criteria for continued therapy (79, 80). Composite endpoints integrating radiographic progression-free survival with spatial and immunologic markers of progression, such as displacement of spatial neighbors or increases in the phagocytic index, should serve as primary endpoints, with overall survival providing a secondary endpoint. Bayesian hierarchical models can facilitate information sharing across biomarker-defined subgroups and improve control of false discovery rates (81, 82). Safety monitoring requires anticipation of overlapping marrow suppression associated with PARPi combined with myeloid-targeting agents. Dose titration rules should follow real-time plasma proteomic changes, including rising CSF1 and IL10 levels, and routine blood counts (83, 84). Data backflow into public repositories, as well as shared blueprint models and anonymous latent-space representations, will improve reproducibility and align external validation with prospective meta-analysis efforts (79, 85). In summary, multi-omics integration enables a closed-loop system of measurement followed by treatment and re-measurement, linking TAM plasticity with combination-therapy selection and allowing iterative improvement of outcomes in patients treated with ADT and PARPi.

## 5 Discussion and future directions

This review integrates current evidence into a unified spatiotemporal model of TAM evolution. We posit that ADT and PARPi initially trigger a ‘recruitment and activation’ phase, offering a transient window for STING-mediated immunity. Over time, however, SASP and the hypoxic bone niche drive a ‘suppressive drift,’ locking macrophages into AR^+^/TREM2^+^ spatial triads that enforce immune exclusion. Durable responses thus require phase-specific interventions: early STING enhancement to ignite immunity, followed by macrophage-targeted therapies (e.g., anti-TREM2 or anti-CD47) to explicitly dismantle these suppressive structural barriers (61). The AR-dependent myeloid circuit, characterized by AR^+^/TREM2^+^ TAM and APOE–TREM2 signaling, provides a coherent explanation for the immune-cold phenotype under androgen pressure and supports ARPI combined with CSF1R or TREM2 inhibition at the peak of monocyte recruitment (4, 41). In PARPi-conditioned tumors, phagocytic thresholds become central determinants of response; combining PARPi with anti-CD47/anti-SIRPα agents or TAM maintenance inhibitors may convert macrophage influx into productive tumor clearance, though adaptive dosing and hematologic monitoring are required (11, 12).

Spatial multi-omics consistently observes TREM2^+^/SPP1^+^ macrophage accumulation within hypoxic, lipid-rich regions of a tumor in proximity to the CAF-vasculature triad, highlighting the necessity to incorporate spatial pharmacology, for example the collapse of TAM-tumor-CAF neighbor clusters, as primary endpoints rather than exploratory biomarkers (4, 14). In practice, tumor genotype, particularly HRR or SPOP status, should anchor composite signatures such as MLCS to guide rational treatment selection, with Bayesian and adaptive designs accelerating the learning–validation cycle (4, 45, 63). Looking forwards, first, the deployment of longitudinal, low-burden sampling methods with plasma proteomics, cfDNA methylation, and minimal IHC/RNA panels for real-time monitoring of innate immune activation even at centers with low technical capacity (86). Second, standardized multimodal AI integration with locked feature dictionaries, stable latent-space references, and transparent model governance to avoid drift and reproducibly synthesize results across platforms (87). And third, expansion of the therapeutic toolbox to include spatiotemporally titratable STING agonists, Fc-engineered or bispecific phagocytic enhancers, and bone-directed or nanocarrier-based formulations designed to re-educate marrow-resident macrophages in the bone niche whilst avoiding global marrow suppression (40, 88). Essentially, the conversion of prostate cancer from an immunologically cold disease into a conditionally hot and controllable condition is a closed-loop: measurement through multi-omics profiling leads to treatment selection based on myeloid and genomic context, then re-measured through example of spatial and functional pharmacodynamics.
